# Sarcoidosis mortality in Sweden: a population-based cohort study

**DOI:** 10.1183/13993003.01815-2017

**Published:** 2018-02-22

**Authors:** Marios Rossides, Susanna Kullberg, Johan Askling, Anders Eklund, Johan Grunewald, Elizabeth V. Arkema

**Affiliations:** 1 Clinical Epidemiology Unit, Dept of Medicine, Solna, Karolinska Institutet, Stockholm, Sweden; 2 Respiratory Medicine Unit, Dept of Medicine, Solna, Karolinska Institutet, Stockholm, Sweden; 3 Center for Molecular Medicine, Karolinska Institutet, Stockholm, Sweden; 4 Dept of Respiratory Medicine, Karolinska University Hospital, Stockholm, Sweden; 5 Dept of Rheumatology, Karolinska University Hospital, Stockholm, Sweden

## Abstract

We aimed to investigate sarcoidosis mortality in a large, population-based cohort, taking into account disease heterogeneity.

Individuals with incident sarcoidosis (n=8207) were identified from the Swedish National Patient Register using International Classification of Disease codes (2003‒2013). In a subset, cases receiving treatment ±3 months from diagnosis were identified from the Prescribed Drug Register. Nonsarcoidosis comparators from the general population were matched to cases 10:1 on birth year, sex and county. Individuals were followed for all-cause death in the Cause of Death Register. Adjusted mortality rates, rate differences and hazard ratios (HRs) were estimated, stratifying by age, sex and treatment status.

The mortality rate was 11.0 per 1000 person-years in sarcoidosis *versus* 6.7 in comparators (rate difference 2.7 per 1000 person-years). The HR for death was 1.61 (95% CI 1.47‒1.76), with no large variation by age or sex. For cases not receiving treatment within the first 3 months, the HR was 1.13 (95% CI 0.94‒1.35). The HR was 2.34 (95% CI 1.99‒2.75) for those receiving treatment.

Individuals with sarcoidosis are at a higher risk of death compared to the general population. For the majority, the increased risk is small. However, patients whose disease leads to treatment around diagnosis have a two-fold increased risk of death. Future interventions should focus on this vulnerable group.

## Abstract 2

**Sarcoidosis is associated with a higher risk of mortality; the risk varies greatly by severity at diagnosis** http://ow.ly/60Ll30h6my8

## Introduction

Sarcoidosis is a granulomatous disease of unknown aetiology affecting primarily the pulmonary and lymphatic systems. The disease is characterised by heterogeneity in terms of presentation and disease course [1]. In some patients, chronic fibrosing disease leads to irreversible organ function decline, but for the majority, the disease self-resolves within a few years after diagnosis [1]. The incidence and prevalence of sarcoidosis in Sweden are among the highest worldwide [2]. Sarcoidosis mortality in Sweden has been considered low and in line with that of other Caucasian populations based on studies from the early 1980s [1, 3], but it is unclear whether this holds true today. An evaluation of how sarcoidosis affects individuals is critical for our understanding of the burden of the disease. It is the first step in identifying strategies for preventing complications of sarcoidosis, and for assessing the effectiveness of current therapeutic management.

Many estimates of sarcoidosis mortality are derived from death certificate data [4–6]. Interpretations from these studies are unreliable due to changes in classification systems over time, but most importantly, attribution of death to a specific cause is often questionable, especially in chronic diseases with several organ manifestations. Prospectively collected, population-based data with sufficient follow-up are needed to determine whether sarcoidosis is associated with long-term consequences. Previous longitudinal studies have yielded conflicting results [7–10]. Two studies showed that the risk of death is more than two-fold higher in patients with sarcoidosis compared to controls [7, 8]. Excess mortality in pulmonary sarcoidosis may only be observed in those with advanced disease stage at diagnosis, as suggested by one Danish study [9]. In contrast, a small study from the Rochester Epidemiology Project in the USA reported no difference in mortality between individuals with sarcoidosis and the general population [10].

We used nationwide Swedish registers to conduct a large, population-based cohort study. The objective of our study was to compare the mortality experience of individuals recently diagnosed with sarcoidosis to that of the general population, overall, by age, sex and by use of treatment around the time point of diagnosis as a marker for more severe sarcoidosis.

## Methods

### Setting and data sources

In Sweden, residents are entitled to universal healthcare coverage that is provided by a tax-funded system of hospitals. The National Patient Register (NPR) captures information on hospitalisations and outpatient (non-primary care) visits. The inpatient component has national coverage since 1987 and the outpatient component since 2001. The Cause of Death Register holds information on the date and causes of death (main and contributory) for almost all residents since 1961. Dispensed prescribed medications in pharmacies across Sweden have been recorded in the Prescribed Drug Register since July 2005. Records in population-based registers can be linked using a unique personal identification number.

### Study design and participants

We conducted a matched cohort study based on data from 2003 to 2013. Information on visits for sarcoidosis was obtained from the NPR using International Classification of Diseases (ICD) codes (ICD-10 D86 including all subcategories, ICD-9/8 135). We identified individuals who had their first ever sarcoidosis visit on January 1, 2003 or later, 2 years after the NPR's outpatient component became available to capture incident sarcoidosis. We required patients to have at least two visits for sarcoidosis >14 days apart and at least one visit at which sarcoidosis was the primary discharge diagnosis. Start of follow-up began on the date they fulfilled the inclusion criteria (index date). To compare sarcoidosis mortality to that of the general population, sarcoidosis cases were individually matched on birth year, sex and county of residence 1:10 to nonsarcoidosis comparators identified from the Total Population Register, which includes all residents. They were required to be resident in Sweden at the time of the case's index date.

To minimise misclassification, the study population was restricted to individuals aged 18‒85 years at start of follow-up. Subjects with a lung cancer or lymphoma diagnosis listed in the Swedish Cancer Register (ICD-7 162, 163, 200‒205) 6 months before or after the first visit for sarcoidosis (or the matched case's first visit for comparators) were excluded. Ethical permission for this study was granted by the regional ethics review board in Stockholm (DNR 2014/230–31).

### Outcome and follow-up

We obtained information on the date of death from the Cause of Death Register. Individuals were followed for all-cause death, first emigration (date obtained from the Total Population Register) or the end of the study (December 31, 2014), whichever occurred first. Following the definition of premature mortality by the Organisation for Economic Co-operation and Development (dx.doi.org/10.1787/193a2829-en), deaths that occurred in individuals aged <70 years were considered premature. During the study period, the average life expectancy at birth in Sweden was 83 years for females and 80 years for males (www.statistikdatabasen.scb.se).

### Covariates and other variables

The date of birth (to calculate age), sex, county of residence (categorised into southern, middle and northern Sweden), and the country of birth (Nordic, non-Nordic) were collected from the Total Population Register. Attained education was obtained from the Education Register (≤9, 10‒12, ≥13  years or missing). Using information from the NPR, we calculated the Charlson comorbidity index using the original Charlson weights [11]. We searched for each comorbidity occurring 1–3 years before the first sarcoidosis visit (see online supplementary table E1 for ICD codes and weights). By restricting the time window for identifying comorbidity, we attempted to capture the load of multimorbidity that was relevant to mortality at baseline and to avoid detection bias affecting the sarcoidosis group.

We hypothesised that individuals in need of treatment at the time of diagnosis represent a group with more severe disease and probably have a different mortality rate. According to national and international guidelines, individuals in need of treatment are those with severe clinical disease (*e.g.* incapacitating symptoms or involvement of vital organs) [1, 12]. Systemic corticosteroids are the first-line treatment and methotrexate or azathioprine are predominantly used as second-line (steroid-sparing) options [12, 13]. We used information in the Prescribed Drug Register to classify individuals as needing treatment at the time of diagnosis if they dispensed at least one prescription of either systemic corticosteroids (ATC H02AB01/02/04/06/07), methotrexate (L01BA01/L04AX03) or azathioprine (L04AX01) within a period of 3 months before or after their first visit for sarcoidosis. As the register was established in July 2005 information was available for a subset of the population entering the cohort starting October 1, 2005 (n=67 408, 75% of the study population).

In addition, using information obtained from the Cause of Death Register, we identified the 10 most common underlying and contributory causes of death in the sarcoidosis group, and calculated their corresponding proportion in the comparator group.

### Statistical analysis

We used Poisson models to estimate age- and sex-adjusted mortality rates and their corresponding 95% confidence intervals in the sarcoidosis and comparator groups. Rate differences were estimated using an additive Poisson model [14] and hazard ratios (HRs) for all-cause death using Cox models adjusted for age, sex and county of residence (model 1), and further for country of birth, education and comorbidity (Charlson index score; model 2). We analysed mortality stratified by age at inclusion, sex and treatment status at diagnosis, and estimated adjusted 1-, 5- and 10-year survival probabilities (model 2) [15]. To estimate the HR for premature mortality we used a fully-adjusted Cox model in which all subjects aged <70 years at inclusion (n=80 686) were followed for all-cause death and right censored at their 70th birthday, first emigration or the end of the study (December 31, 2014).

We tested the robustness of the HR against the potential confounding of current smoking (data for which we did not have) using probabilistic bias analysis methods [16]. Smoking is associated with a lower risk of developing sarcoidosis and a higher risk of death [17, 18]. In simulations, we made the following informed assumptions for three bias parameters based on Swedish health surveys (The Public Health Agency of Sweden; www.folkhalsomyndigheten.se) and previously published literature: smoking prevalence in sarcoidosis (range 9‒16%) and comparators (21‒27%), and a relative risk of death due to smoking of 2.6 (see online supplementary table E2 for detailed definitions) [17–19]. We simulated another dataset to test possible misclassification of our register-based definition for sarcoidosis. Study participants had their sarcoidosis status reclassified assuming positive predictive values 50‒70% and negative predictive values 98‒100%. The effect of misclassification and confounding on the HR was then tested together in a single simulation. Simulation confidence intervals were obtained using bootstrapping techniques. We used SAS (version 9.4; SAS Institute, Cary, NC, USA) and Stata (version 14.2; StataCorp, College Station, TX, USA) for data management and statistical analysis.

## Results

We identified 8207 individuals with sarcoidosis and 81 119 matched general population comparators between 2003 and 2013. Table 1 shows the demographic and clinical characteristics of the two groups at baseline. The mean±sd age at inclusion was 49±14.4 years and 56% were male. Individuals with sarcoidosis had more comorbid conditions than comparators (mean±sd Charlson comorbidity index score 0.24±0.86 *versus* 0.13±0.60, p<0.001 from t-test). 12% of individuals with sarcoidosis and 7% of general population comparators had at least one comorbidity. 6191 (42%) out of 2599 individuals with sarcoidosis entering the cohort starting October 1, 2005 received treatment at the time of diagnosis. Baseline characteristics of the two subgroups of individuals with sarcoidosis and of matched comparators are presented in online supplementary table E3.

**TABLE 1:** Baseline demographic and clinical characteristics of individuals with sarcoidosis and matched general population comparators

	**Sarcoidosis**	**General population**
**Subjects**	8207	81 119
**Age at inclusion years**	49±14.4	49±14.4
**Sex**		
Female	3613 (44)	35 765 (44)
Male	4594 (56)	45 354 (56)
**Region of residence**		
Northern Sweden	1118 (14)	11 118 (14)
Middle Sweden	3285 (40)	32 329 (40)
Southern Sweden	3804 (46)	37 672 (46)
**Country of birth**		
Nordic	7407 (90)	71 268 (88)
Non-Nordic	800 (10)	9851 (12)
**Years of education**		
≤9	1627 (20)	16 274 (20)
10–12	4058 (50)	37 672 (46)
≥13	2435 (30)	26 256 (32)
Missing	87 (1)	917 (1)
**Charlson comorbidity index score**	0.24±0.86	0.13±0.60

Data are presented as n, mean±sd or n (%).

During a similar median follow-up of 5.9 years (interquartile range 3.4‒8.7 years), 528 deaths occurred in the sarcoidosis group and 3204 in the comparator group (table 2). Sarcoidosis was the predominant underlying or contributory cause of death among the 445 individuals with sarcoidosis who died during 2003‒2013 (n=134, 30%; online supplementary table E4). On average, individuals with sarcoidosis died 2 years younger than their general population comparators (mean±sd age at death 69.5±12.6 years *versus* 71.5±12.5, p=0.001 from t-test).

**TABLE 2:** All-cause mortality in individuals with sarcoidosis *versus* matched general population comparators

	**Sarcoidosis**		**General population**		**Adjusted mortality rate** **difference (95% CI)^¶^**	**Hazard ratio** **(95% CI)**	
	**Deaths per** **person-years**	**Adjusted mortality** **rate (95% CI)^#^**	**Deaths per** **person-years**	**Adjusted mortality** **rate (95% CI)^#^**		**Model 1^+^**	**Model 2^§^**
**Subjects n**	8207		81 119				
**Overall**	528/47 810	11.0 (10.1–12.0)	3204/479 200	6.7 (6.5–6.9)	2.7 (2.3–3.1)	1.74 (1.59–1.91)	1.61 (1.47–1.76)
**Age at inclusion years**							
18–29	2/4049	0.9 (0.6–1.3)	24/40 689	0.5 (0.3–0.8)	-0.1 (-0.8–0.6)	0.84 (0.20–3.56)	0.69 (0.16–2.96)
30–39	25/12 464	1.6 (1.3–1.9)	109/121 763	0.9 (0.8–1.1)	1.1 (0.3–1.9)	2.24 (1.45–3.46)	1.62 (1.02–2.56)
40–49	41/11 080	3.0 (2.5–3.5)	192/110 249	1.8 (1.5–2.0)	1.9 (1.5–2.2)	2.13 (1.52–2.98)	2.03 (1.45–2.85)
50–59	80/9606	8.6 (7.6–9.6)	476/96 636	5.0 (4.6–5.5)	2.8 (2.0–3.6)	1.70 (1.34–2.15)	1.54 (1.21–1.96)
60–69	156/6999	21.0 (18.8–23.1)	838/71 388	12.3 (11.5–13.0)	9.0 (6.8–11.3)	1.92 (1.62–2.28)	1.65 (1.38–1.96)
70–85	224/3614	72.5 (65.8–79.1)	1565/38 476	42.5 (40.3–44.5)	18.0 (11.2–25.4)	1.61 (1.39–1.84)	1.52 (1.32–1.75)
**Sex**							
Female	260/20 973	11.5 (10.5–12.5)	1659/211 050	6.7 (6.4–7.0)	3.2 (2.5–3.9)	1.64 (1.44–1.87)	1.55 (1.36–1.77)
Male	268/26 838	15.2 (13.9–16.6)	1545/268 150	8.9 (8.4–9.3)	2.6 (2.1–3.1)	1.85 (1.64–2.11)	1.68 (1.47–1.91)

^#^: per 1000 person-years, estimated using Poisson models mutually adjusted for age and sex; ^¶^: estimated using additive Poisson models adjusted for exponential age and sex, region of residence, country of birth, education and comorbidity (Charlson comorbidity index score; continuous); the model did not converge for age groups 18–29 and 30–39 years, hence estimates are unadjusted rate differences; ^+^: estimated using Cox models adjusted for age (continuous), sex and region of residence; ^§^: estimated using Cox models further adjusted for country of birth, education and comorbidity (Charlson comorbidity index score; continuous).

**FIGURE 1:**
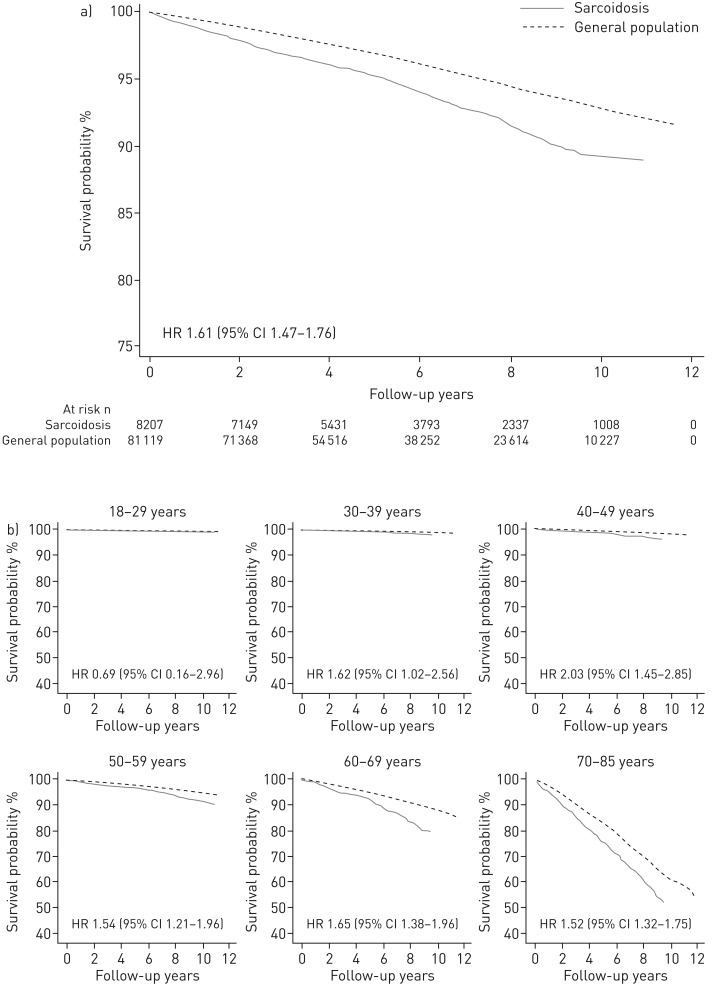
Adjusted survival functions of individuals with incident sarcoidosis and general population comparators a) overall and b) by age at inclusion.

The age- and sex-adjusted mortality rate was 11.0 per 1000 person-years in sarcoidosis (95% CI 10.1‒12.0) and 6.7 per 1000 person-years in comparators (95% CI 6.5‒6.9; table 2). There were three more deaths per 1000 person-years in the sarcoidosis group compared to the comparators (95% CI 2.4‒3.2). Mortality rates were higher in individuals with sarcoidosis compared to comparators irrespective of age or sex, except in the youngest individuals (18‒39 years), for whom death was uncommon. The overall adjusted 1-, 5- and 10-year survival probabilities were 98.9%, 95.4% and 89.4% for sarcoidosis, respectively, and 99.6%, 96.9% and 92.9% for the comparators (figure 1a).

**FIGURE 2:**
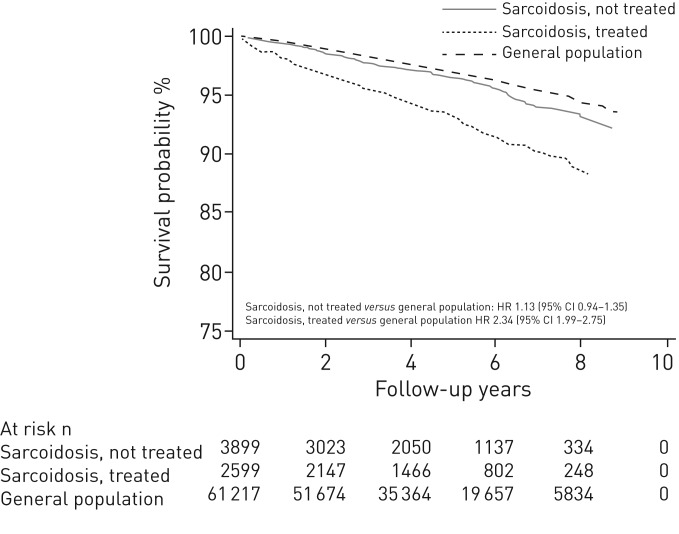
Adjusted survival functions of individuals with incident sarcoidosis compared to the general population, stratified by treatment status.

The fully adjusted HR for all-cause death was 1.61 (95% CI 1.47‒1.76; table 2). The risk for premature death was 64% higher in sarcoidosis compared to comparators (HR 1.64, 95% CI 1.43‒1.89). No large variation in the relative risk of death by age or sex was observed. For individuals treated at diagnosis the HR was 2.34 (95% CI 1.99‒2.75) *versus* 1.13 (95% CI 0.94‒1.35) for those who did not receive treatment at diagnosis (online supplementary table E5). Survival functions stratified by age and treatment status at diagnosis are depicted in figure 1b and figure 2, respectively.

The bias analysis showed that the conventional HR (from the main analysis) was relatively robust even under extreme bias assumptions (table 3). The HR for death did not differ greatly (1.66, 95% bootstrapped interval 1.40‒1.93) when we accounted for potential confounding by current smoking and for misclassification of our sarcoidosis definition in a single simulation.

**TABLE 3:** Probabilistic bias analysis accounting for unmeasured confounding by current smoking and sarcoidosis misclassification

	**Hazard ratio** **(95% CI)**
**Conventional analysis**	
Random error	1.61 (1.47–1.76)
**Sensitivity analyses^#^**	
Unmeasured confounding by current smoking	
Systematic error	1.74 (1.71–1.78)
Systematic error and random error	1.74 (1.60–1.93)
Misclassification of sarcoidosis definition	
Systematic error	1.54 (1.42–1.66)
Systematic error and random error	1.54 (1.31–1.79)
Unmeasured confounding by current smoking and	
misclassification of sarcoidosis definition	
Systematic error	1.66 (1.52–1.78)
Systematic error and random error	1.66 (1.40–1.93)

^#^: estimated by simulations based on predetermined assumptions for bias parameters (see online supplementary material for their specification). Confidence intervals are simulation intervals for systematic error analyses and bootstrapped intervals for systematic error and random error analyses.

## Discussion

In this study, individuals with sarcoidosis had a higher mortality rate compared to matched comparators from the general population. After adjusting for relevant confounders, individuals with sarcoidosis had a 62% higher risk for all-cause death compared to the general population and an excess of three deaths per 1000 person-years were related to sarcoidosis. In addition, sarcoidosis was associated with a greater risk of premature death (death at <70 years of age). Despite the overall differences in mortality, our results suggest that individuals with sarcoidosis who did not receive treatment at diagnosis had only a marginally greater risk of death. However, the risk was increased more than two-fold for those treated, representing more severe disease at diagnosis.

The results of this study are in line with most, but not all previous investigations. A small study from the USA of mainly Caucasian incident cases reported no association between sarcoidosis and mortality (standardised mortality ratio 0.90) [10]. However, it should be noted that the mortality rate in the sarcoidosis group was similar to the one in our study (13.3 per 1000 person-years) [10], hence the difference on the ratio scale may be attributable to the choice of comparators. Nonetheless, our results were similar to a study from the UK (1991‒2003; mortality rate 14 per 1000 person-years, HR 2.09) despite differences in case ascertainment (they used primary care data to identify sarcoidosis) [7].

In the report by Tukey *et al*. [8], sarcoidosis in the Black Women's Health Study was associated with a 2.4-fold higher risk of death, which is higher than our overall estimate. Two factors are likely to have contributed to the observed difference. Tukey *et al*. included cases at various disease stages in their cohort (incident, prevalent and deaths due to sarcoidosis), whereas we limited inclusion to newly diagnosed cases. Another explanation could be that individuals of African American descent have worse prognosis, with previous studies suggesting that sarcoidosis is diagnosed earlier, more extrapulmonary organs are involved and the rate of sarcoidosis-related hospitalisations is higher in black individuals compared to white individuals [20, 21].

Mortality estimates in this study, both absolute and relative, are generally lower than those reported in previous investigations for sarcoidosis [1]. This might reflect improved diagnosis and survival compared to previous decades, or might be a feature of our population, in which prognosis might be better [1]. Moreover, despite the later (10 years) peak in disease incidence in females [2], the HR for death was similar for both sexes. This observation is consistent with previous longitudinal studies [7, 9] and contradicts some studies utilising death certificate datasets that indicated prominently higher mortality in females [4, 5, 22]. Our study showed that sarcoidosis is associated with an excess of three deaths per 1000 person-years in our population, an estimate comparable to other inflammatory diseases such as rheumatoid arthritis [23].

To address our concern that any potential greater risk for death in sarcoidosis compared to the general population was possibly driven by a smaller group of patients with more severe disease, we defined a proxy for severe disease based on dispensation of sarcoidosis-related treatments around the time of disease diagnosis. We showed that the risk of death in individuals treated was more than twice that of the general population, whereas the risk was only slightly increased for patients who did not require such treatment. This finding, while in line with a single-centre study from the 1990s in pulmonary sarcoidosis patients [9], should be cautiously interpreted, as short-term corticosteroid treatment courses are not free from adverse effects and the role of early administration of such therapies remains controversial for some patient subgroups [24, 25]. In addition, there might be other factors affecting the decision to initiate treatment, which may have had some influence on the observed results.

Our analysis of death certificate data of individuals with sarcoidosis in our cohort showed that the disease is the most commonly mentioned underlying and/or contributing cause of death in these individuals. However, sarcoidosis was listed in only 30% of the deaths in the sarcoidosis cohort. Factors such as organ engagement (*e.g.* cardiac sarcoidosis), time since diagnosis, reporting practices or sarcoidosis misclassification in this study might influence this observation. Therefore, it should be noted that while studies utilising death certificate datasets provide an interesting snapshot of disease burden at death, they are methodologically limited to account for the whole picture of disease course, as they capture only a subset of sarcoidosis patients [26, 27].

Our study has some limitations. A register-based definition was used to identify incident sarcoidosis cases and therefore some misclassification is expected. We did not have any information on primary care visits. However, the majority of patients with sarcoidosis in Sweden are diagnosed in specialist care, therefore we do not believe we have missed very many cases. Furthermore, the HR for death did not change significantly when subjected to testing for misclassification under extreme terms. Lastly, due to the lack of clinical patient information, we were unable to investigate mortality by disease phenotype or contrast our medication-based proxy for severity with other disease severity indices. The use of treatment as a proxy for disease severity might have also led to some misclassification. Future studies are warranted to investigate mortality in well-defined patient subgroups with different disease phenotypes.

A major strength of our study is the use of high-quality registers with excellent coverage of the entire Swedish population. They provided a population-based sample and the largest study to date to examine mortality longitudinally. Interpretations from this study are generalisable to populations with similar standards of healthcare. Complete follow-up was possible and relevant confounding factors (except lifestyle factors such as smoking) were available for all individuals. Moreover, two potential sources of bias were quantitatively addressed using advanced analysis techniques, indicating the robustness of our results.

In conclusion, we have demonstrated that the overall risk of death in individuals with sarcoidosis is greater compared to the general population. However, it varies considerably with disease severity. It was only slightly increased in the majority of patients who did not receive treatment at the time of diagnosis, but was increased two-fold for individuals who received corticosteroids, methotrexate or azathioprine at the time of diagnosis, probably due to more severe disease. This subset of patients with a higher risk of poor outcomes should be prioritised for future interventions aiming to reduce the burden of sarcoidosis, and the effectiveness of treatments should be evaluated.

## Supplementary material

10.1183/13993003.01815-2017.Supp1 **Please note:** supplementary material is not edited by the Editorial Office, and is uploaded as it has been supplied by the author.
Supplementary tables ERJ-01815-2017_Supplement

## Disclosures

10.1183/13993003.01815-2017.Supp2 E.V. Arkema ERJ-01815-2017_Arkema
