# Supplementary material for: Sarcoidosis mortality in Sweden: a population-based cohort study
Source: Eur Respir J. 2018 Feb 22;51(2):1701815. doi: 10.1183/13993003.01815-2017 (PMC5886843; doi:10.1183/13993003.01815-2017)
Supplement: Supplementary file 1 [file ERJ-01815-2017_Supplement.pdf]

SUPPLEMENTARY

Sarcoidosis mortality in Sweden: a population-based cohort study

Marios Rossides, Susanna Kullberg, Johan Askling, Anders Eklund, Johan Grunewald, Elizabeth V. Arkema

Contents

Table E1. ICD-10 codes used to identify comorbidities in the National Patient Register and weights used to calculate the Charlson Comorbidity Index score.

Table E2. Distributions of bias parameters used in probabilistic bias analyses to account for unmeasured confounding by current smoking and misclassification of sarcoidosis definition.

Table E3. Baseline characteristics of individuals with sarcoidosis who received treatment or not at diagnosis and their matched general population comparators entering the cohort after Oct 1, 2005.

Table E4. Ten most common underlying and contributory causes of death in individuals with sarcoidosis and their corresponding proportions in the general population (2003–2013).

Table E5. All-cause mortality in individuals with sarcoidosis compared to matched general population comparators entering the cohort Oct 1, 2005, overall and stratified by treatment status.

Table E1. ICD-10 codes used to identify comorbidities in the National Patient Register and weights used to calculate the Charlson Comorbidity Index score.

Comorbidity	ICD-10 codes*	Weight
Myocardial infarction	I21–I22, I25.2	1
Congestive heart failure	I09.9, I11.0, I13.0, I13.2, I25.5, I42.0, I42.5–I42.9, I43, I50, P29.0	1
Peripheral vascular disease	I70–I71, I73.1, I73.8, I73.9, I77.1, I79.0, I79.2, K55.1, K55.8–K55.9, Z95.8–Z95.9	1
Cerebrovascular disease	G45–G46, H34.0, I60–I69	1
Dementia	F00–F03, F05.1, G30, G31.1	1
Chronic pulmonary disease	I27.8, I27.9, J40–J47, J60–J67, J68.4, J70.1, J70.3	1
Rheumatic disease	M05–M06, M31.5, M32–M34, M35.1, M35.3, M36.0	1
Peptic ulcer disease	K25–K28	1
Mild liver disease	B18, K70.0–K70.3, K70.9, K71.3–K71.5, K71.7, K73–K74, K76.0, K76.2–K76.4, K76.8–K76.9, Z94.4	1
Diabetes without chronic complications	E10.0–E10.1, E10.6, E10.8–E10.9, E11.0–E11.1, E11.6, E11.8–E11.9, E12.0–E12.1, E12.6, E12.8–E12.9, E13.0–E13.1, E13.6, E13.8–E13.9, E14.0–E14.1, E14.6, E14.8–E14.9	1
Diabetes with chronic complications	E10.2–E10.5, E10.7, E11.2–E11.5, E11.7, E12.2–E12.5, E12.7, E13.2–E13.5, E13.7, E14.2–E14.5, E14.7	2
Hemiplegia or paraplegia	G04.1, G11.4, G80.1, G80.2, G81–G82, G83.0–G83.4, G83.9	2
Renal disease	I12.0, I13.1, N03.2–N03.7, N05.2–N05.7, N18–N19, N25.0, Z49.0–Z49.2, Z94.0, Z99.2	2
Any malignancy, including lymphoma and leukaemia, except malignant skin neoplasms	C00–C26, C30–C34, C37–C41, C43, C45–C58, C60–C76, C81–C85, C88, C90–C97	2
Moderate or severe liver disease	I85.0, I85.9, I86.4, I98.2, K70.4, K71.1, K72.1, K72.9, K76.5, K76.6, K76.7	3
Metastatic solid tumour	C77–C80	6
AIDS/HIV infection	B20–B22, B24	6

* The Swedish version of the ICD-10 classification system came into effect in 1997.
The codes were taken from Quan *et al.*, 2005, Med Care, and the weights from Charlson *et al.*, 1987, J Chronic Dis.

Table E2. Distributions of bias parameters used in probabilistic bias analyses.

Bias parameter	Type of distribution	Minimum	Lower mode	Upper mode	Maximum
Unmeasured confounding by smoking					
Prevalence in sarcoidosis	Trapezoidal	0.09	0.10	0.15	0.16
Prevalence in controls	Trapezoidal	0.10	0.11	0.16	0.17
Additive prevalence in all-cause death due to smoking*	Uniform	0.12	NA	NA	0.16
Sarcoidosis definition misclassification					
Positive predictive value	Uniform	0.50	NA	NA	0.70
Negative predictive value	Uniform	0.98	NA	NA	100.0

NA = not applicable.

Data are proportions.

* Corresponds to a relative risk of all-cause death due to smoking of 2.60.

Table E3. Baseline characteristics of individuals with sarcoidosis who received treatment or not at diagnosis and their matched general population comparators entering the cohort after Oct 1, 2005.

	**Sarcoidosis, treated* (n=2599)**	**Sarcoidosis, not treated* (n=3592)**	**General population (n=61217)**
Mean age at inclusion (SD), years	49.8 (14.8)	49.8 (14.2)	49.8 (14.4)
Sex, n (%)			
Female	1087 (42)	1634 (45)	26938 (44)
Male	1512 (58)	1958 (55)	34279 (56)
Region of residence, n (%)			
Northern Sweden	342 (13)	518 (14)	8539 (14)
Middle Sweden	915 (35)	1548 (43)	24253 (40)
Southern Sweden	1342 (52)	1526 (42)	28425 (46)
Country of birth, n (%)			
Nordic	2360 (91)	3195 (89)	53380 (88)
Non-Nordic	239 (9)	397 (11)	7837 (13)
Years of education, n (%)			
≤9	532 (20)	659 (18)	11933 (19)
10–12	1353 (52)	1752 (49)	28312 (46)
≥13	695 (27)	1136 (32)	20243 (33)
Missing	19 (1)	45 (1)	729 (1)
Mean Charlson Comorbidity Index score (SD)	0.26 (0.82)	0.26 (0.95)	0.14 (0.62)

* Defined as the dispensation of at least one prescription for oral corticosteroids, methotrexate, or azathioprine in the Prescribed Drug Register, three months before and after the first sarcoidosis visit.

Table E4. Ten most common underlying or contributory causes of death in individuals with sarcoidosis and their corresponding proportions in the general population comparators group.

No.	ICD-10 code	Disease/disorder's name	Sarcoidosis (n=445)	General population (n=2606)
**Underlying causes of death**				
1	D86	Sarcoidosis	10.3	0.0
2	I21	Acute myocardial infarction	8.1	8.1
3	I25	Chronic ischemic heart disease	4.7	6.4
4	C34	Malignant neoplasm of bronchus and lung	4.3	5.3
5	C18	Malignant neoplasm of colon	2.9	3.1
6	C56	Malignant neoplasm of ovary	2.9	1.4
7	C25	Malignant neoplasm of pancreas	2.7	2.6
8	E14	Unspecified diabetes mellitus	2.7	1.4
9	J84	Other interstitial pulmonary diseases	2.5	0.7
10	C50	Malignant neoplasm of breast	2.3	3.4
**Contributory causes of death**				
1	D86	Sarcoidosis	30.1	0.0
2	I50	Heart failure	20.0	16.1
3	C79	Secondary malignant neoplasm of other and unspecified sites	12.4	12.7
4	I25	Chronic ischemic heart disease	12.4	13.7
5	E14	Unspecified diabetes mellitus	11.5	6.5
6	I21	Acute myocardial infarction	10.6	10.0
7	J18	Pneumonia, organism unspecified	10.3	8.3
8	I46	Cardiac arrest	8.8	6.9
9	I10	Essential (primary) hypertension	8.5	9.5
10	J84	Other interstitial pulmonary diseases	7.2	1.0

Data are percentage of total number of deaths.

Table E5. All-cause mortality in individuals with sarcoidosis compared to matched general population comparators entering the cohort Oct 1, 2005, overall and stratified by the receipt of treatment at diagnosis.

Analysis	n	Deaths/ person-years	Hazard ratio (95% CI)*
**Overall**			
Sarcoidosis	6547	370/31377	1.64 (1.46–1.83)
General population	64691	2158/314493	1.00 [Reference]
**Treated at diagnosis†**			
Sarcoidosis, treated	2599	183/11908	2.34 (1.99–2.75)
General population	25726	826/121293	1.00 [Reference]
Sarcoidosis, not treated	3592	136/16704	1.13 (0.94–1.35)
General population	35491	1033/165286	1.00 [Reference]

* Estimated by Cox models adjusted for age, sex, and region of residence, country of birth, education, and comorbidity (Charlson Index score).
† Defined as the dispensation of at least one prescription for oral corticosteroids, methotrexate, or azathioprine in the Prescribed Drug Register, three months before and after the first sarcoidosis visit.
